# Qualidade de Vida em Longo Prazo e Desfechos após Internação por COVID-19 no Brasil: Protocolo do Estudo Pós-COVID Brasil 1

**DOI:** 10.36660/abc.20230378

**Published:** 2023-11-13

**Authors:** Geraldine Trott, Fernando Luis Scolari, Marciane Maria Rover, Mariana Motta Dias da Silva, Denise de Souza, Rosa da Rosa Minho dos Santos, Raíne Fogliati de Carli Schardosim, Gabriela Soares Rech, Juliana de Mesquita, Gabriel Pozza Estivalete, Hellen Jordan Martins Freitas, Carolina Rothmann Itaqui, Amanda Christina Kozesinski-Nakatani, Andreia Biolo, Milena Soriano Marcolino, Bruna Brandão Barreto, Paulo Roberto Schvartzman, Ana Carolina Peçanha Antonio, Caroline Cabral Robinson, Maicon Falavigna, Carisi Anne Polanczyk, Regis Goulart Rosa

**Affiliations:** 1 Escritório de Projetos de Pesquisa Hospital Moinhos de Vento Porto Alegre RS Brasil Escritório de Projetos de Pesquisa – Hospital Moinhos de Vento , Porto Alegre , RS – Brasil; 2 Divisão de Cardiologia Hospital Moinhos de Vento Porto Alegre RS Brasil Divisão de Cardiologia do Hospital Moinhos de Vento , Porto Alegre , RS – Brasil; 3 Unidade de Terapia Intensiva Hospital Santa Casa de Curitiba Curitiba PR Brasil Unidade de Terapia Intensiva – Hospital Santa Casa de Curitiba , Curitiba , PR – Brasil; 4 Instituto Nacional de Avaliação de Tecnologias em Saúde Universidade Federal do Rio Grande do Sul Porto Alegre RS Brasil Instituto Nacional de Avaliação de Tecnologias em Saúde – Universidade Federal do Rio Grande do Sul , Porto Alegre , RS – Brasil; 5 Departamento de Medicina Interna Faculdade de Medicina Universidade Federal de Minas Gerais Belo Horizonte MG Brasil Departamento de Medicina Interna da Faculdade de Medicina da Universidade Federal de Minas Gerais , Belo Horizonte , MG – Brasil; 6 Departamento de Medicina Interna e Apoio Diagnóstico Faculdade de Medicina da Bahia Universidade Federal da Bahia Salvador BA Brasil Departamento de Medicina Interna e Apoio Diagnóstico , Faculdade de Medicina da Bahia , Universidade Federal da Bahia , Salvador , BA – Brasil; 7 Unidade de Terapia Intensiva Hospital da Mulher – Maria Luzia Costa dos Santos Salvador BA Brasil Unidade de Terapia Intensiva – Hospital da Mulher – Maria Luzia Costa dos Santos , Salvador , BA – Brasil; 8 Unidade de Terapia Intensiva Hospital de Clínicas de Porto Alegre Porto Alegre RS Brasil Unidade de Terapia Intensiva – Hospital de Clínicas de Porto Alegre , Porto Alegre , RS – Brasil; 9 Unidade de Pesquisa Inova Medical Porto Alegre RS Brasil Unidade de Pesquisa – Inova Medical , Porto Alegre , RS – Brasil; 10 Serviço de Medicina Interna Hospital Moinhos de Vento Porto Alegre RS Brasil Serviço de Medicina Interna – Hospital Moinhos de Vento , Porto Alegre , RS – Brasil

**Keywords:** Síndrome Pós-COVID-19 Aguda, Qualidade de Vida, Tempo

## Abstract

**Fundamento:**

O impacto em longo prazo da hospitalização por COVID-19 sobre a saúde física, mental e cognitiva dos pacientes requer mais investigação.

**Objetivos:**

Este artigo visa avaliar os fatores associados com a qualidade de vida e desfechos cardiovasculares e não cardiovasculares 12 meses após a internação hospitalar por COVID-19.

**Métodos:**

Este estudo multicêntrico prospectivo pretende incluir 611 pacientes internados por COVID-19 (NCT05165979). Entrevistas telefônicas centralizadas estão programadas para ocorrer em três, seis, nove e 12 meses após a alta hospitalar. O desfecho primário é definido como o escore de utilidade de qualidade de vida relacionada à saúde avaliada pelo questionário EuroQol-5D-3L (EQ-5D-3L) aos 12 meses. Desfechos secundários são definidos como o EQ-5D-3L aos três, seis e nove meses, retorno ao trabalho ou à escola, sintomas persistentes, novas incapacidades em atividades instrumentais diárias, déficit cognitivo, ansiedade, depressão, e sintomas de transtorno do estresse pós-traumático, eventos cardiovasculares maiores, reinternação, e mortalidade por todas as causas aos três, seis, nove e 12 meses após a infecção pelo SARS-CoV-2. Um valor de p<0,05 será considerado estatisticamente significativo para as análises.

**Resultados:**

O desfecho primário será apresentado como frequência do escore EQ-5D-3L 12 meses após a internação por COVID-19. Uma subanálise para identificar possíveis associações das variáveis independentes com desfechos do estudo será apresentada.

**Conclusão:**

Este estudo determinará o impacto da COVID-19 sobre a qualidade de vida e de desfechos cardiovasculares e não cardiovasculares de pacientes internados 12 meses após a alta, e fornecerá novas informações ao sistema público de saúde no Brasil.

**Figura Central:**
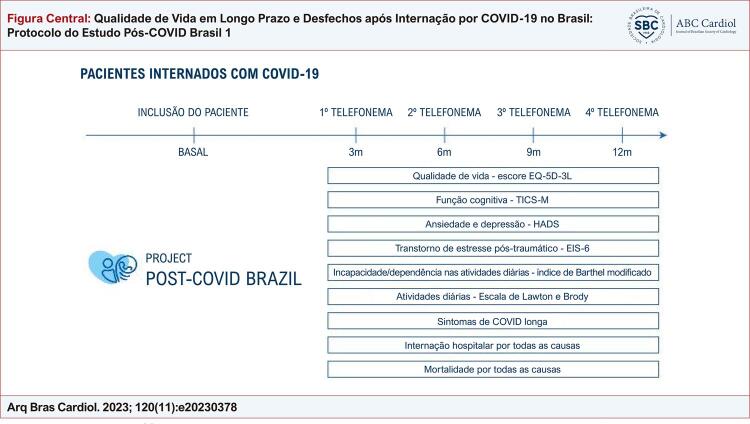
: Qualidade de Vida em Longo Prazo e Desfechos após Internação por COVID-19 no Brasil: Protocolo do Estudo Pós-COVID Brasil 1

Delineamento do estudo pós-COVID Brasil.

## Introdução

A pandemia da COVID-19 teve um grave impacto em todo o mundo, com mais de 676 milhões de casos registrados e mais de seis milhões de mortes até 2023. ^1^ Só no Brasil, mais de 37 milhões de casos haviam sido registrados até maio de 2023, com mais de 699 mil mortes. ^2 , 3^ Esses números alarmantes representam uma crise na saúde mundial, de proporções sem precedentes. Além disso, COVID-19 foi associada a sintomas de longo prazo em uma proporção significativa de pacientes infectados. ^4^ A Organização Mundial da Saúde (OMS) reconheceu a presença da COVID longa, mas evidências sobre seu impacto sobre a saúde cognitiva, física e mental em longo prazo são ainda limitadas. A COVID longa é caracterizada por sintomas persistentes por um mínimo de dois meses sem outra explicação causal. ^4^ Esses sintomas podem ocorrer em 10-20% dos pacientes e têm um impacto negativo sobre a qualidade de vida desses indivíduos. De fato, poucos pacientes relataram terem se recuperado completamente e estarem assintomáticos 60 dias após a COVID-19 aguda, enquanto 44% relataram piora na qualidade de vida. ^5^ Após internação por COVID-19, pelo menos 34% dos indivíduos continuam a apresentar sintomas seis semanas após a alta. ^6^ Assim, estudos abordando a frequência de pacientes que apresentam sintomas de COVID longa e piora na qualidade de vida são necessários.

Embora eventos cardiovasculares tenham sido relatados durante a fase aguda da COVID-19, dados recentes sugerem que complicações como insuficiência cardíaca, fibrilação atrial, e pericardite podem ocorrer até 30 dias após a infecção aguda. ^7^ Um número significativo de pacientes com COVID longa apresenta dispneia e outros sintomas cardiopulmonares, e pelo menos 50% dos pacientes encaminhados aos ambulatórios relatam tais sintomas. ^8^ Ainda, a incidência de insuficiência cardíaca nos pacientes com COVID-19 prévia parece ser quase o dobro da incidência em pacientes que não tiveram a doença, mesmo nove meses após a fase aguda. ^9^ Estudos também identificaram uma associação entre COVID-19 e disfunção endotelial, aumentando o risco de aterosclerose. ^10 , 11^ Pacientes com condições cardiovasculares pré-existentes podem ter apresentado descompensação até 30 dias após a infecção aguda. ^12^ Elevação nos níveis aumentados de troponina durante a fase aguda da COVID-19 foi associada a complicações cardiovasculares em longo prazo, sendo um indicador de lesão miocárdica causada pelo SARS-CoV-2. ^13^ Este conjunto de evidências sugere que a COVID-19 pode aumentar o risco cardiovascular a longo prazo. ^14^

Este estudo teve como objetivo avaliar o impacto em longo prazo da internação por COVID-19 sobre a qualidade de vida dos pacientes 12 meses após a alta. Objetivos secundários do estudo incluíram avaliar a mortalidade global, eventos cardiovasculares maiores, reinternação precoce e tardia, retorno ao trabalho ou aos estudos, persistência dos sintomas, novas incapacidades nas atividades instrumentais da vida diária, disfunção cognitiva, bem como ansiedade, depressão, e sintomas de transtorno de estresse pós-traumático em três, seis, nove e 12 meses após infecção moderada a grave pelo SARS-CoV-2.

## Métodos

### Delineamento do estudo

Este é um estudo prospectivo multicêntrico do tipo coorte de pacientes com COVID-19 internados. O recrutamento dos pacientes ocorre durante a primeira internação, e o acompanhamento por meio de contato por telefone em três, seis, nove, e 12 meses após a alta. A Figura Central ilustra o delineamento do estudo.

### Elegibilidade dos pacientes

Serão considerados elegíveis pacientes com idade ≥ 18 anos e sintomas clínicos compatíveis com infecção por SARS-CoV-2 e resultado positivo para COVID-19 por transcrição reversa seguido da reação em cadeia da polimerase (PCR-RT) ou teste de antígeno. Pacientes com expectativa de vida de menos de três meses devido à alguma comorbidade, pacientes com deficiência de comunicação verbal (tais como afasia, déficits cognitivos, ou não falantes da língua portuguesa), pacientes sem apoio familiar, pacientes sem número de telefone, pacientes ausentes, pacientes que retiraram o consentimento, e pacientes que já haviam sido incluídos no estudo serão excluídos. A Tabela 1 resume os critérios de eligibilidade do estudo.

**Tabela 1:** – Critérios de inclusão e exclusão do estudo Pós-COVID Brasil

Critérios de inclusão	
Idade ≥ 18 anos	
Internação hospitalar por COVID-19 por período igual ou superior a 48 horas	
Teste RT-PCR positivo para SARS-CoV-2 com *swab* nasofaríngeo ou teste de antígeno positivo para SARS-CoV-2 com *swab* nasofaríngeo até 14 dias antes da primeira internação	
Pelo menos um dos seguintes sintomas 14 dias antes da primeira internação: Febre (> 38 °C), tosse, espirros, dispneia, saturação periférica de oxigênio (<95%) no ar ambiente, perda ou alteração do olfato (anosmia) ou paladar (ageusia), coriza, dor de garganta, dor de cabeça, mialgia, dor na articulação, e diarreia	
Critérios de exclusão	Racional
Expectativa de vida < três meses por comorbidade subjacente segundo avaliação pela equipe médica	Incapacidade de completar os primeiros três meses de seguimento após a internação por COVID-19
Ausência de suporte familiar em um paciente com disfunção de linguagem ou de comunicação (afasia, déficit cognitivo, não ser falante da língua portuguesa)	A entrevista por telefone exige que o paciente responda questões sobre sintomas, qualidade de vida, e desfechos. A incapacidade de fazer isso resultaria em seguimento incompleto
Ausência de número de telefone para contato	Todos os questionários serão administrados por entrevista telefônica
Retirada do consentimento	A inclusão dos participantes sem consentimento resultaria em questões éticas
Participantes incluídos previamente no estudo	Dupla entrada no estudo levaria a um viés de seleção
Óbito durante a primeira internação	Incapacidade de completar o acompanhamento

### Locais do estudo

O recrutamento dos participantes para este estudo será realizado em hospitais de referência para o tratamento de COVID-19 no Brasil. A seleção dos hospitais participantes será feita pelo centro coordenador com base nos seguintes critérios:

- O hospital deve ser um centro de referência para o tratamento de COVID-19.
- O hospital deve ter uma unidade de terapia intensiva com capacidade de 10 ou mais leitos para o tratamento de COVID-19. O RT-PCR para o diagnóstico de COVID-19 deve estar disponível no hospital.
- O hospital deve concordar em participar do estudo assinando um acordo de cooperação.
- Em cada centro, os pacientes consecutivos que preencherem os critérios de eligibilidade serão recrutados e acompanhados aos três, seis, nove, e 12 meses após a inclusão por um *call center* localizado no Hospital Moinhos de Vento.

## Desfechos

### Desfecho primário

O desfecho primário é definido como escore de utilidade da qualidade de vida relacionada à saúde, que será avaliada 12 meses após a primeira internação por COVID-19 usando o questionário EuroQol com cinco dimensões e três níveis (EQ-5D-3L). O EQ-5D-3L consiste em um escore descritivo composto por cinco dimensões que avalia mobilidade, autocuidado, atividades diárias, dor/desconforto e ansiedade/depressão, com três níveis de gravidade: nenhum problema, alguns problemas, e problemas extremos. Além disso, uma Escala Analógica Visual (EAV) para autoavaliação do estado geral de saúde será obtida dos pacientes. O EQ-5D-3L varia na população brasileira de -0,17 (indicando o pior estado de saúde, com problemas extremos em todas as dimensões) a 1 (indicando o melhor estado de saúde, sem problemas em nenhuma dimensão). ^14^ A mínima diferença clinicamente importante estimada para o EQ-5D-3L é 0,03, ^15^ com um valor médio de 0,82 para a população brasileira. ^16^ Em caso de óbito durante o seguimento, o paciente receberá um escore de zero nas demais avaliações após o evento.

### Desfechos secundários

Os desfechos secundários serão avaliados em quatro momentos: aos três, seis, nove, e 12 meses após a alta hospitalar. Esses incluem: 1 – Qualidade de vida avaliada pelo EQ-5D-3L (em um, três, seis e nove meses); 2- Morte antes de 12 meses após a alta; 3 – Eventos cardiovasculares maiores: morte cardiovascular, infarto agudo do miocárdio não fatal, e acidente vascular cerebral não fatal 12 meses após a alta; 4 – incidência de reinternação precoce não programada (20 dias) ou tardia (31-180 dias); 5 – prevalência de sintomas em longo prazo: dispneia, tosse, fadiga, fraqueza muscular, dor torácica, dor nas articulações, anosmia, queda de cabelo, dificuldade de se concentrar, e distúrbios do sono; 6 - disfunção cognitiva avaliada pela entrevista telefônica modificada para avaliação do estado cognitivo (TICS-M, *Telephone Interview for Cognitive Status-modified* ); ^17^ 7 – sintomas de ansiedade e depressão estimados pela escala HADS ( *Hospital Anxiety and Depression Scale* ); ^18^ 8 - transtorno de estresse pós-traumático de acordo com a escala de impacto do evento IES-6 ( *Impact of Event Scale-6* ); ^19^ 9 – estado físico funcional avaliado usando-se o índice de Barthel modificado; ^20^ 10 – novas deficiências em atividades instrumentais diárias avaliadas usando a escala de Lawton & Brody (qualquer deficiência, mudando de independente para parcialmente dependente ou parcialmente dependente para totalmente dependente, em pelo menos um dos seguintes domínios: uso do telefone, transporte, compras, responsabilidade por seus medicamentos, e capacidade em lidar com finanças) em relação a um mês antes da COVID-19; ^21^ 11 – retorno ao trabalho ou à escola; e 12 - reinfecção sintomática pelo SARS-CoV-2 (definida como recorrência de sintomas de COVID-19 e infecção confirmada por RT-PCR ou antígeno positivo para SARS-CoV-2 mais de 90 dias após a infecção primária).

### Variáveis prognósticas

O estudo avaliará cinco conjuntos de variáveis que podem estar associadas com o prognóstico dos pacientes internados com COVID-19: A) variáveis sociodemográficas como idade, sexo, educação, e renda; B) comorbidades tais como doenças cardíacas, obesidade, doença respiratória, doença renal crônica, imunossupressão, câncer, doenças neurológicas, doença hepática, doenças hematológicas, e doença mental; C) status de vacinação contra COVID-19; D) gravidade da COVID-19, avaliada pela pior condição durante a internação de acordo com a classificação da OMS – 1. Internação hospitalar sem suporte de oxigênio; 2. Internação hospitalar com suporte de oxigênio com dispositivo de baixo fluxo (cânula nasal, máscara facial simples); 3. Internação hospitalar com suporte de oxigênio por ventilação não invasiva ou cânula nasal de alto fluxo; 4. Internação hospitalar por ventilação mecânica ou Oxigenação por Membrana Extracorpórea (ECMO); e E) Exames laboratoriais e de imagem durante a internação a critério de cada centro. Os exames laboratoriais incluem medidas de proteína C reativa, dímeros D, troponina, peptídeo natriurético cerebral, e contagem de linfócitos. O estudo de imagens inclui tomografia computadorizada do tórax e ecocardiograma.

### Acompanhamento

O período de acompanhamento iniciará no dia da alta hospitalar. O primeiro telefonema de seguimento será feito aos três meses (±15 dias) após a alta. Os telefonemas serão realizados a todos os indivíduos aos três, seis, nove e 12 meses após a alta hospitalar. O seguimento é realizado por um *call center* centralizado com pesquisadores treinados no Hospital Moinhos de Vento. Todos os telefonemas serão gravados e registrados em um banco de dados eletrônico. As chamadas serão realizadas em turnos e em dias diferentes, e serão usados todos os números fornecidos pelo centro de recrutamento. Se o número fornecido não estiver correto, um novo número de telefone será solicitado do centro de recrutamento. Dez tentativas consecutivas sem sucesso serão consideradas como perda de seguimento. Os participantes responderão perguntas de um questionário estruturado com questões sobre estado de vida, reinternações, eventos cardiovasculares maiores, retorno ao trabalho, sintomas em longo prazo (tais como dispneia, tosse, fadiga, fraqueza muscular, desconforto no peito, anosmia, perda de cabelo, dificuldade de se concentrar, e insônia), estado físico funcional avaliado pelo índice de Barthel modificado, novas incapacidades nas atividades diárias avaliadas pela escala de Lawton e Brody, disfunção cognitiva avaliada pelo TICS-m, ansiedade e depressão avaliadas pelo HADS, síndrome do estresse pós-traumático avaliada pelo IES-6, e qualidade de vida avaliada pelo EQ-5D-3L. Com exceção do HADS, IES-6, e TICS-m, todos os demais instrumentos de avaliação poderão ser respondidos por um membro da família ou representante legal.

### Qualidade e segurança dos dados

A coleta de dados será realizada usando um formulário eletrônico de relato de caso, que pode ser acessado via smartphones, tablets ou computadores pessoais. A coleta e o manejo de dados digitais oferecem muitas vantagens, incluindo padronização, confiabilidade, e segurança dos dados. A ferramenta que será usada para o gerenciamento dos dados será o REDCap (Research Electronic Data Capture - https://www.redcapbrasil.com.br/). O acesso a essa plataforma é permitido por meio de um nome de usuário e uma senha únicos e intransferíveis fornecidos a cada pesquisador. Cada membro da equipe recebe permissão específica para acesso aos dados com base no seu papel no estudo determinado pelo investigador principal.

Para assegurar a qualidade dos dados, vários procedimentos de segurança serão implementados incluindo:

1. Todos os pesquisadores receberão treinamento sobre a coleta de dados e procedimentos do estudo;
2. Todos os pesquisadores terão acesso ao centro coordenador para questionamentos e resolução de problemas;
3. Todo o gerenciamento dos dados será feito de acordo com a legislação nacional de proteção de dados (lei 13.709 de 14 de agosto de 2018).
4. Acesso ao banco de dados será restrito a nomes de usuários e senhas únicas e intransferíveis;
5. Uma cópia de segurança dos dados será rotineiramente feita a cada 24 horas usando um protocolo automatizado. A extração dos dados será realizada com anonimização de dados, para a análise da consistência dos dados, monitoramento remoto dos dados, desenvolvimento de dados derivados, e análise estatística.
6. A limpeza de dados será realizada periodicamente, e os pesquisadores serão notificados de qualquer inconsistência e correção dos dados.
7. Todas as chamadas telefônicas serão gravadas e auditadas para consistência dos dados. Os arquivos dos áudios serão armazenados em servidor digital anônimo com o mesmo protocolo de segurança do conjunto de dados principal; o acesso aos arquivos de áudio será restrito a nomes de usuários e senhas únicos e intransferíveis fornecidos a cada pesquisador.
8. O centro coordenador revisará relatórios mensais sobre o rastreamento, critérios de inclusão, e acompanhamento dos pacientes, consistência dos dados, e completude dos dados e, se necessário, imediatamente tomará medidas para resolver qualquer problema.
9. Procedimentos estatísticos serão realizados ao longo do estudo para possíveis detectar fraudes.

### Tamanho da amostra

Nós calculamos o tamanho amostral em 556 pacientes usando o método PEAR. ^22^ Essa abordagem considerou uma eficácia de precisão de 0,8, inclusão de três preditores na regressão logística, e um tamanho do efeito de 0,26. Para considerar potenciais perdas de seguimento, acrescentamos um adicional de 10% de inclusão, resultando em um tamanho amostral final de 611 pacientes. Nossa análise assumiu um poder de 80%, e um nível alfa bilateral de 0,05.

### Gerenciamento de dados faltantes

Para os dados faltantes das escalas HADS e IES-6, serão considerados as médias das variáveis faltantes na mesma subescala, se pelo menos metade dela estiver completa.

### Análise estatística

A normalidade dos dados será avaliada usando histogramas e o teste de Shapiro-Wilk. As variáveis contínuas serão apresentadas em média ± desvio padrão ou mediana e intervalo interquartil de acordo com a distribuição dos dados. As variáveis categóricas serão apresentadas como contagem total e frequência relativa. Mortalidade por todas as causas, eventos cardiovasculares maiores, reinternação e retorno ao trabalho ou à escola serão apresentados como taxas de incidência. Estado físico funcional, sintomas de ansiedade e depressão, sintomas de síndrome de estresse pós-traumático, e função cognitiva serão descritos como taxas de prevalência, usando pontos de corte clínicos relevantes. A associação entre variáveis dependentes e desfechos será avaliada usando equações de estimativas generalizadas, ajustando-se por efeitos agrupados e medidas repetidas. A multicolinearidade será avaliada usando o fator de inflação da variância. Um nível de significância de 0,05 será usado para todas as comparações. Todas as análises serão realizadas usando o programa R versão 4.2.2 ( *R Foundation for Statistical Computing* ).

### Aspectos éticos e de disseminação

#### Aprovação ética

O estudo foi registrado no ClinicalTrials.gov (NCT05165979), aprovado pelo comitê de ética institucional (CAAE, 54665321.6.1001.5330) e pelos comitês de ética de todos os centros participantes, e está de acordo com a resolução 466/2012 do Conselho Nacional de Saúde. Consentimento por escrito será obtido de cada participante no momento do recrutamento. O estudo foi planejado e conduzido de acordo com a resolução 466 de 12 de dezembro de 2012 do Conselho Nacional da Saúde, ^23^ e com o adendo E6 das diretrizes de boas práticas clínicas (segunda revisão) do *International Council for Harmonization* , (ICH-GCP). ^24^ O estudo também está de acordo com a Lei Geral de Proteção de Dados Pessoais (número 13709 de 14 de agosto de 2018).

#### Procedimentos de consentimento

De acordo com as diretrizes e padrões regulatórios em pesquisa envolvendo seres humanos estabelecidos na resolução 466/2012 do Conselho Nacional da Saúde, um termo de consentimento detalhado será entregue a cada participante no momento do convite ao estudo. O termo será escrito em linguagem clara e inclusiva, descrevendo os objetivos, a metodologia, e informações sobre a coleta e o armazenamento de dados. O investigador responsável ou outros investigadores explicará os procedimentos e os riscos do estudo, e os benefícios ao participante. Os participantes ou seus representantes legais serão informados que todos os indivíduos incluídos serão voluntários que poderão retirar o consentimento em qualquer momento sem que isso afete seu tratamento. Ainda, os participantes serão informados que todos os registros poderão ser acessados por autoridades de saúde local e pelo centro coordenador, sem violação da confidencialidade, conforme permitido pelos regulamentos nacionais em pesquisa. Os participantes e os representantes legais terão tempo suficiente para ler o termo de consentimento e fazer perguntas antes de assiná-lo. O termo será assinado pelo participante (ou seu representante legal) e pelo pesquisador, e cada um ficará com uma cópia.

#### Confidencialidade

Para assegurar a confidencialidade das informações, serão tomadas medidas rígidas. O acesso às informações pessoais será limitado aos pesquisadores, e os nomes e as informações confidenciais dos participantes não serão fornecidos a nenhum terceiro. Todas as informações serão consideradas confidenciais e usadas exclusivamente para fins de estudo. O acesso ao documento será restrito àqueles com permissão, e o banco de dados eletrônico será acessado por meio de nomes de usuários e senhas únicos. Para assegurar a confidencialidade, os resultados serão publicados como dados agrupados, o que previne a identificação dos participantes individuais. Esses dados serão publicados somente para fins científicos e acadêmicos. Todos os dados serão gerenciados de acordo com a Lei Geral de Proteção de Dados Pessoais (número 13709 de 14 de agosto de 2018) para proteger a privacidade dos participantes.

#### Disseminação

Para assegurar transparência e disseminação dos resultados do estudo, os pesquisadores apresentarão os resultados completos do estudo em reuniões e congressos científicos. Além disso, os resultados serão publicados em revistas com revisão por pares, seguindo-se as diretrizes e as regras estabelecidas pelo *International Committee of Medical Journal Editors* . A escolha da revista para publicação será feita pelo comitê diretivo, com base na qualidade e na importância da revista, bem como no potencial impacto dos resultados do estudo sobre a comunidade científica. Os pesquisadores irão assegurar que todas as publicações declarem a fonte de financiamento e o papel dos financiadores se o caso. Finalmente, os pesquisadores assegurarão que todas as publicações reflitam com precisão os resultados obtidos do estudo e tomarão medidas para resolver quaisquer conflitos de interesse.

#### Compartilhamento de dados

Os autores encorajam o compartilhamento de dados e acesso aberto para promover a transparência e a reprodutibilidade da pesquisa. Solicitações para acesso aos dados devem ser submetidas ao autor de correspondência e serão avaliadas pelo comitê diretivo. Qualquer compartilhamento de dados seguirá a Lei Geral de Proteção de Dados Pessoais (número 13709 de 14 de agosto de 2018) e regulamentos legais e éticos aplicáveis. Em caso de compartilhamento de dados, serão tomadas medidas apropriadas para assegurar a confidencialidade das informações pessoais.

## Discussão

O estudo tem como objetivo abordar uma importante lacuna na literatura investigando o impacto da internação por COVID-19 leve a moderada sobre a qualidade de vida, conduzindo um estudo observacional rigoroso com uma grande amostra e longo período de seguimento. Enquanto há plausibilidade científica de que a gravidade da COVID-19 esteja associada com qualidade de vida em longo prazo, atualmente, os dados disponíveis são limitados. Estudos anteriores foram limitados pelo tamanho amostral pequeno e curtos períodos de seguimento, como aqueles com apenas três meses de acompanhamento. ^25^ O recente estudo observacional multicêntrico PHOSP-COVID avaliou pacientes sobreviventes após a internação por COVID-19 cinco meses após a alta e encontrou que a doença cardiovascular foi a comorbidade mais comum (42%). ^26^ Os autores do estudo também observaram que somente 28% dos participantes se consideraram totalmente recuperados, e os preditores para a falta de recuperação ou persistência dos sintomas foram sexo feminino, etnia branca, duas ou mais comorbidades, e gravidade da COVID-19 (baseada na escala da OMS, que inclui ventilação mecânica ou necessidade de ECMO durante a internação hospitalar). ^24^ O Coalition VII, um estudo prospectivo multicêntrico do tipo coorte incluiu 1508 pacientes internados com suspeita ou diagnóstico confirmado de COVID-19, mostrou que os pacientes que necessitaram de ventilação mecânica durante a internação apresentaram uma qualidade de vida em um ano mais baixa que aqueles que não necessitaram. ^14^ Conduzindo um estudo observacional rigoroso com uma grande amostra e um longo período de acompanhamento, cobrindo o período pós-vacinação e diferentes variantes predominantes do SARS-CoV-2, o presente estudo tem como objetivo elucidar o impacto em longo prazo da internação por COVID-19 moderada a grave sobre a qualidade de vida dos pacientes.

Dados populacionais do Brasil indicam um aumento na mortalidade cardiovascular durante o primeiro ano da pandemia da COVID-19. ^27^ Isso provavelmente se deve ao vírus afetando o sistema cardiovascular por vários mecanismos, tais como disfunção microvascular, desequilíbrio entre demanda e suprimento de oxigênio, lesão miocárdica direta, e cardiotoxicidade. ^28^ Esses mecanismos na fase aguda da COVID-19 pode contribuir para complicações cardiovasculares em longo prazo. ^10 , 28^ Portanto, é crucial avaliar e monitorar os pacientes quanto a sequelas cardiovasculares após a infecção aguda pelo SARS-CoV-2 para identificar os indivíduos em risco e promover tratamento adequado para doença cardíaca secundária, incluindo insuficiência cardíaca. ^9 , 12 , 29^ No entanto, a carga real da COVID longa sobre a saúde cardiovascular continua desconhecida, e mais pesquisa é necessária para determinar seu impacto. Nosso estudo tem como objetivo destacar não só a prevalência dos sintomas cardiovasculares, como seus desfechos.

O estudo tem vários pontos fortes, incluindo seu delineamento multicêntrico prospectivo, com um tamanho amostral grande de sobreviventes de COVID-19 moderada a grave, acompanhados por 12 meses. No entanto, algumas limitações devem ser reconhecidas. Por exemplo, a associação de sintomas em longo prazo com COVID-19 prévia pode estar sujeita a viés por vários fatores, tais como subjetividade na gravidade da doença, percepções pessoais sobre a saúde, e fatores socioculturais relacionados à pandemia. Além disso, como não serão realizados exames ou avaliações complementares para excluir outras causas desses sintomas, eles podem ser erroneamente atribuídos à infecção prévia pelo SARS-CoV-2.

Deve-se destacar que o planejamento e o protocolo do estudo foram completados em novembro de 2021, com o recrutamento agendado para começar em dezembro de 2021. No entanto, o comitê diretivo reconsiderou a duração do estudo com base no número de casos na população e o número de participantes incluídos. Os telefonemas de seguimento dos pacientes iniciaram-se em março de 2022, e se espera que o acompanhamento de um ano termine até abril de 2024.
