# Supplementary material for: Qualidade de Vida em Longo Prazo e Desfechos após Internação por COVID-19 no Brasil: Protocolo do Estudo Pós-COVID Brasil 1
Source: Arq Bras Cardiol. 2023 Nov 13;120(11):e20230378. [Article in Portuguese] doi: 10.36660/abc.20230378 (PMC10697686; doi:10.36660/abc.20230378)
Supplement: Translation

# Translation: Long-term Health-Related Quality of Life and Outcomes after Hospitalization for COVID-19 in Brazil: Post-COVID Brazil 1 Study Protocol

**Authors:** Geraldine Trott, Fernando Luis Scolari, Marciane Maria Rover, Mariana Motta Dias da Silva, Denise de Souza, Rosa da Rosa Minho dos Santos, Raíne Fogliati de Carli Schardosim, Gabriela Soares Rech, Juliana de Mesquita, Gabriel Pozza Estivalete, Hellen Jordan Martins Freitas, Carolina Rothmann Itaqui, Amanda Christina Kozesinski-Nakatani, Andreia Biolo, Milena Soriano Marcolino, Bruna Brandão Barreto, Paulo Roberto Schvartzman, Ana Carolina Peçanha Antonio, Caroline Cabral Robinson, Maicon Falavigna, Carisi Anne Polanczyk, Regis Goulart Rosa

**Affiliations:** 1 Brazil Escritório de Projetos de Pesquisa – Hospital Moinhos de Vento, Porto Alegre, RS – Brazil; 2 Brazil Divisão de Cardiologia do Hospital Moinhos de Vento, Porto Alegre, RS – Brazil; 3 Brazil Unidade de Terapia Intensiva – Hospital Santa Casa de Curitiba, Curitiba, PR – Brazil; 4 Brazil Instituto Nacional de Avaliação de Tecnologias em Saúde – Universidade Federal do Rio Grande do Sul, Porto Alegre, RS – Brazil; 5 Brazil Departamento de Medicina Interna da Faculdade de Medicina da Universidade Federal de Minas Gerais, Belo Horizonte, MG – Brazil; 6 Brazil Departamento de Medicina Interna e Apoio Diagnóstico, Faculdade de Medicina da Bahia, Universidade Federal da Bahia, Salvador, BA – Brazil; 7 Brazil Unidade de Terapia Intensiva – Hospital da Mulher – Maria Luzia Costa dos Santos, Salvador, BA – Brazil; 8 Brazil Unidade de Terapia Intensiva – Hospital de Clínicas de Porto Alegre, Porto Alegre, RS – Brazil; 9 Brazil Unidade de Pesquisa – Inova Medical, Porto Alegre, RS – Brazil; 10 Brazil Serviço de Medicina Interna – Hospital Moinhos de Vento, Porto Alegre, RS – Brazil

**Keywords:** Post-acute COVID-19 Syndrome, Quality of Life, Time

## Abstract

**Background:**

The long-term impact of hospitalization for COVID-19 on patients’ physical, mental, and cognitive health still needs further assessment.

**Objectives:**

This study aims to evaluate factors associated with quality of life and cardiovascular and non-cardiovascular outcomes 12 months after hospitalization for COVID-19.

**Methods:**

This prospective multicenter study intends to enroll 611 patients hospitalized due to COVID-19 (NCT05165979). Centralized telephone interviews are scheduled to occur at three, six, nine, and 12 months after hospital discharge. The primary endpoint is defined as the health-related quality-of-life utility score assessed by the EuroQol-5D-3L (EQ-5D-3L) questionnaire at 12 months. Secondary endpoints are defined as the EQ-5D-3L at three, six and nine months, return to work or education, persistent symptoms, new disabilities in instrumental activities of daily living, cognitive impairment, anxiety, depression, and post-traumatic stress symptoms, major cardiovascular events, rehospitalization, as well as all-cause mortality at 3, 6, 9, and 12 months after SARS-CoV-2 infection. A p-value <0.05 will be assumed as statistically significant for all analyses.

**Results:**

The primary endpoint will be presented as the frequency of the EQ-5D-3L score 12 months after COVID-19 hospitalization. A sub-analysis to identify possible associations of independent variables with study outcomes will be presented.

**Conclusions:**

This study will determine the impact of COVID-19 on the quality of life and cardiovascular and non-cardiovascular outcomes of hospitalized patients 12 months after discharge providing insights to the public health system in Brazil.

**Central Illustration:** : Long-term Health-Related Quality of Life and Outcomes after Hospitalization for COVID-19 in Brazil: Post-COVID Brazil 1 Study Protocol

Post-COVID Brazil study design.

## Introduction

The COVID-19 pandemic had a severe impact worldwide, with more than 676 million reported cases and over six million deaths until 2023.^1^In Brazil alone, more than 37 million cases had been reported by May 2023, with more than 699,000 deaths.^2 , 3^These staggering numbers represent a global health crisis of unprecedented proportions. Additionally, COVID-19 has been linked to long-term symptoms in a significant proportion of infected patients.^4^The World Health Organization (WHO) has acknowledged the presence of long COVID, but evidence of its impact on cognitive, physical, and mental health in the long term is still limited. Long COVID is characterized by persistent symptoms for a minimum of two months without other explanation.^4^These symptoms may occur in 10% to 20% of patients and have a negative impact on the quality of life of these individuals. In fact, a few patients have reported being fully recovered and asymptomatic 60 days after acute COVID-19, whereas 44% have reported impaired quality of life.^5^After COVID-19 hospitalization, at least 34% of individuals continue to experience symptoms six weeks after discharge.^6^Therefore, studies are still needed to fully address the rate of patients experiencing long-COVID symptoms and quality-of-life impairment.

Although cardiovascular events have been reported during the acute phase of COVID-19, recent data suggest that complications such as heart failure, atrial fibrillation, and pericarditis may occur up to 30 days after the acute infection.^7^A significant number of patients with long COVID experience dyspnea and other cardiopulmonary symptoms, and at least 50% of patients referred to outpatient clinics report such symptoms.^8^Furthermore, the incidence of heart failure in patients with prior COVID-19 appears to be almost double that of patients without it, even nine months after the acute phase.^9^Studies have also identified an association between COVID-19 and endothelial dysfunction, increasing the risk of atherosclerosis.^10 , 11^Patients with preexisting cardiovascular conditions may experience decompensation up to 30 days after the acute infection.^12^Troponin elevation during the acute phase of COVID-19 has been linked to long-term cardiovascular complications, being an indicator of myocardial injury caused by SARS-CoV-2.^13^This body of evidence suggests that COVID-19 may increase cardiovascular risk in the long term.^14^

This study aims to evaluate the long-term impact of COVID-19 hospitalization on patients’ quality of life 12 months after discharge. Secondary objectives include assessing overall mortality, major cardiovascular events, early and late rehospitalization, return to work or education, symptom persistence, new disabilities in instrumental activities of daily living, cognitive impairment, as well as anxiety, depression, and post-traumatic stress symptoms at three, six, nine and 12 months following moderate-to-severe SARS-CoV-2 infection.

## Methods

### Study design

This is a prospective multicenter cohort study of hospitalized patients with COVID-19. Enrollment is conducted during the index hospitalization, and follow-up is performed through structured centralized telephone calls at three, six, nine, and 12 months after discharge. Central figure illustrates the study design.

### Patient eligibility

Patients ≥18 years of age with clinical symptoms compatible with SARS-CoV-2 infection and with a positive COVID-19 reverse transcription polymerase chain reaction (RT-PCR) or antigen test will be considered eligible. Patients with a life expectancy of less than three months due to any underlying comorbidity, those with verbal communication disabilities (such as aphasia, cognitive deficits, or non-Portuguese speakers), patients without family support, those who did not have a telephone, those who are absent, those who withdraw consent, or those who had been previously included in this study will be excluded. Table 1 summarizes the study’s eligibility criteria.

**Table 1:** – Inclusion and exclusion criteria of the Post-COVID Brazil study

Inclusion criteria	
	≥18 years of age		
	Hospitalization for COVID-19 ≥48 hours		
	Positive RT-PCR test for SARS-CoV-2 with nasopharyngeal swab or positive antigen test for SARS-CoV-2 with nasopharyngeal swab up to 14 days prior to index hospitalization		
	At least one of the following symptoms 14 days prior to index hospitalization: Fever (>38 °C), cough, sneezing, dyspnea, peripheral oxygen saturation (<95%) on room air, impairment of the sense of smell (anosmia) or taste (ageusia), coryza, sore throat, headache, myalgia, joint pain, and diarrhea		
Exclusion criteria	Rationale
Life expectancy <three months due to underlying comorbidity at the discretion of the medical team	Inability to complete the first three-month follow-up period after the COVID-19 hospitalization
Absence of familial support in a patient with language/communication disability (aphasia, cognitive deficit, non-Portuguese speaker)	Telephone follow-up requires the patient to answer questions about symptoms, quality of life, and outcomes. Inability to do so would result in incomplete follow-up
No telephone available for contact	All questionnaires will be administered via telephone interview
Withdrawal of consent	The inclusion of participants without consent would result in ethical issues
Participants previously included in the study	Double entry in the study would lead to selection bias
Death during index hospitalization	Inability to complete follow-up

### Study sites

The recruitment of participants for this study will take place at COVID-19 referral hospitals in Brazil. Selection of the participating hospitals will be made by the coordinating center based on the following criteria:

- The hospital must be a referral center for COVID-19 care.
- The hospital must have an intensive care unit with a capacity of 10 or more beds for COVID-19 treatment. RT-PCR for the diagnosis of COVID-19 must be available in the hospital.
- The hospital must agree to participate in the study by signing a cooperation agreement.
- In each center, consecutive patients who meet the study eligibility criteria will be recruited and followed up at three, six, nine, and 12 months after inclusion through a local call center located at Hospital Moinhos de Vento.

### Outcomes

#### Primary outcome

The study primary outcome is defined as the health-related quality-of-life utility score, which will be assessed 12 months after the index COVID-19 hospitalization using the EuroQol 5-dimension 3-level (EQ-5D-3L) questionnaire. The EQ-5D-3L is a 5-dimension descriptive score that assesses mobility, self-care, daily activities, pain/discomfort, and anxiety/depression symptoms, with three levels for each dimension: no problems, some problems, and extreme problems. In addition, a visual analog scale (VAS) for self-rating of overall health status will be obtained from all patients. The EQ-5D-3L ranges in the Brazilian population from -0.17 (indicating the worst health status, with extreme problems in all dimensions) to 1 (indicating optimal health status, with no problems in any dimension).^14^The minimal clinically important difference in the EQ-5D-3L has been estimated at 0.03,^15^with a mean value of 0.82 for the Brazilian population.^16^In the event of a patient death during follow-up, the patient will be given a score of 0 in all remaining assessments after the event.

#### Secondary outcomes

The secondary outcomes will be assessed four times: at three, six, nine, and 12 months after hospital discharge. They include: 1 – Quality of life assessed by the EQ-5D-3L (at 3, 6, and 9 months); 2 – Death up to 12 months after discharge; 3 – Major cardiovascular events: cardiovascular death, non-fatal acute myocardial infarction, and non-fatal stroke 12 months after discharge; 4 – Incidence of non-planned early (30 days) or late (31-180 days) rehospitalization; 5 – Prevalence of long-term symptoms: dyspnea, cough, fatigue, muscle weakness, chest discomfort, joint pain, anosmia, hair loss, difficulty concentrating, and sleep disorder; 6 – Cognitive impairment assessed using the Telephone Interview for Cognitive Status-modified (TICS-m);^17^7 – Anxiety and depression symptoms estimated by the Hospital Anxiety and Depression Scale (HADS);^18^8 – Post-traumatic stress disorder according to the Impact of Event Scale-6 (IES-6);^19^9 – Functional physical status assessed by the modified Barthel index;^20^10 – New disabilities in instrumental activities of daily living assessed by the Lawton & Brody scale (any impairment, moving from independent to partially dependent or from partially dependent to totally dependent, in at least one of the following domains: telephone use, transportation, shopping, responsibility for own medications, and ability to handle finances) relative to one month before COVID-19;^21^11 – Return to work or education; and 12 – Symptomatic SARS-CoV-2 reinfection (defined as the recurrence of COVID-19-like symptoms confirmed by a positive RT-PCR or antigen test for SARS-CoV-2 more than 90 days after primary infection).

#### Prognostic variables

This study will assess five sets of variables that may be associated with the prognosis of hospitalized patients with COVID-19: A) Social and demographic variables, including age, sex, education, and income; B) Comorbidities, such as cardiovascular diseases, obesity, respiratory illness, chronic kidney disease, immunosuppression, cancer, neurologic disorders, hepatic disease, hematologic diseases, and mental illness; C) COVID-19 vaccination status; D) COVID-19 severity, assessed by the worst condition during hospitalization according to the WHO classification (1. Hospitalization without oxygen support; 2. Hospitalization requiring oxygen support with low-flow device [nasal cannula, simple face mask]; 3. Hospitalization requiring oxygen support by noninvasive ventilation or high-flow nasal cannula; 4. Hospitalization requiring oxygen support by invasive ventilation or extracorporeal membrane oxygenation [ECMO]); and E) Laboratory and imaging studies during hospitalization at the discretion of each center. Laboratory tests include C-reactive protein, D-dimer, troponin, brain natriuretic peptide, and lymphocyte count. Imaging study includes chest computed tomography and echocardiogram.

#### Follow-up

The first follow-up call will be scheduled for three months following discharge, with a window of ±15 days. Telephone calls will be made to all individuals at three, six, nine, and 12 months after hospital discharge. The follow-up will be conducted by a centralized call center with trained researchers at the Hospital Moinhos de Vento. All calls will be recorded and registered in an electronic database. The calls will be made on different shifts and days of the week, and all available numbers provided by the enrolling center will be used. If the provided number is incorrect, a new telephone number will be requested from the enrolling center. Ten consecutive unsuccessful attempts will be defined as loss to telephone follow-up. Participants will answered a structured questionnaire with questions on vital status, rehospitalizations, major cardiovascular events, return to work, long-term symptoms (such as dyspnea, cough, fatigue, muscle weakness, chest discomfort, joint pain, anosmia, hair loss, difficulty concentrating, and insomnia), functional physical status assessed by the modified Barthel index, new disabilities in instrumental activities of daily living assessed by the Lawton & Brody scale, cognitive impairment assessed by the TICS-m, anxiety and depression assessed by the HADS, post-traumatic stress disorder assessed by the IES-6, and quality of life assessed by the EQ-5D-3L. Except for the HADS, IES-6, and TICS-m, all other evaluation instruments can be answered by a family member or a legal representative with a close relationship to the participant.

#### Data quality and safety

Data collection will be performed using an electronic case report form, which can be accessed via smartphones, tablets, or personal computers. Digital data collection and management offer several advantages, including data standardization, reliability, and safety. The tool used for data management will be the REDCap (Research Electronic Data Capture - https://www.redcapbrasil.com.br/). Access to this platform is granted through a unique and non-transferable username and password assigned to each research member. Each member of the research team is assigned specific permission for data access based on their proposed study role as determined by the principal investigator.

To ensure data quality, several safety procedures will be implemented, including:

1. All researchers will receive specific training on data collection and study procedures.
2. Sub-investigators will have access to the coordinating center for questioning and troubleshooting.
3. All data management will be conducted in compliance with the Brazilian General Data Protection Regulation (Law No. 13709 of August 14, 2018).
4. Dataset access will be restricted to unique and non-transferable usernames and passwords.
5. Dataset backups will be routinely performed every 24 hours using an automated protocol. Data extraction will be performed with data anonymization for data consistency analysis, remote data monitoring, derived data development, and statistical analysis.
6. Inconsistent data cleaning will be conducted periodically, and sub-investigators will be notified of any data inconsistencies and corrections.
7. All telephone calls will be recorded and audited for data consistency. Audio files will be stored in an anonymous digital server with the same security protocol as the main dataset, and file access will be restricted to unique and non-transferable usernames and passwords assigned to research staff.
8. The coordinating center will review monthly reports on screening, patient inclusions, follow-up, consistencies, and completeness of data. If necessary, immediate action will be taken to resolve any issues.
9. Statistical techniques will be employed to detect data fraud during the study.

#### Sample size

We calculated the required sample size to be 556 patients using the PEAR method.^22^This approach considered a precision efficacy of 0.8, the inclusion of three predictors in the logistic regression, and an effect size of 0.26. To account for potential loss to follow-up, we added an additional 10% inclusion, resulting in a final sample size of 611 patients. Our analysis assumed a power of 80% and a two-sided alpha level of 0.05.

#### Missing data management

Missing data for the HADS and IES-6 scales will be imputed by using the mean values of the missing variables within the same subscale if at least half of the subscale is complete.

#### Statistical analysis

Data normality will be assessed using histograms and the Shapiro-Wilk test. Continuous variables will be reported as mean ± standard deviation (SD) or median and interquartile range (IQR) according to data distribution. Categorical variables will be presented as total count and relative frequency. All-cause mortality, major cardiovascular events, rehospitalization and return to work or education will be reported as incidence rate ratios. Functional physical status, anxiety and depression symptoms, post-traumatic stress symptoms, and cognitive function will be reported as prevalence rate ratios, using clinically relevant cutoffs. The association between dependent variables and outcomes will be assessed using generalized estimating equations, adjusting for cluster effects and repeated measures. Multicollinearity will be evaluated using the variance inflation factor. A significance level of 0.05 will be used for all comparisons. All analyses will be performed using R version 4.2.2 (R Foundation for Statistical Computing).

## Ethics and dissemination

### Ethical approval

The study was registered at ClinicalTrials.gov (NCT05165979), approved by the institutional ethics committee (CAAE, 54665321.6.1001.5330) and by the research ethics committees of all participating centers, and complies with the Brazilian National Health Council Resolution 466/2012. Written informed consent will be obtained from all participants included in the study at the time of enrollment.

The study was planned and conducted in accordance with the Brazilian National Health Council Resolution 466 of December 12, 2012,^23^and the Good Clinical Practice Guidelines, amendment 6 – 2nd revision of the International Council for Harmonization (ICH-GCP).^24^The study also adhered to the Brazilian General Personal Data Protection Law (No. 13709 of August 14, 2018).

### Consent procedures

According to the Guidelines and Regulating Standards in Human Research established by the Brazilian National Health Council Resolution 466/2012, a comprehensive consent form will be provided to the participant during the study invitation process. The form will be written in plain and inclusive language, outlining the study’s objectives, methodology, data collection, and storage processes. The responsible investigator or sub-investigator will explain the study’s procedures and risks, and benefits to the participant. Participants or their legal representatives will be informed that all included individuals are volunteers and that they can withdraw consent at any time without affecting their medical care. Additionally, participants will be informed that local health authorities and the coordinating center may access all records without violating confidentiality, as permitted by national research regulations. Participants and legal representatives will be given adequate time to read the consent form and ask questions before signing it. Both the participant or legal representative and the investigator sign the consent form, and one legal copy will be provided to each party.

### Confidentiality

To ensure the confidentiality of participant information, strict measures will be taken. Access to personal information will be limited to study investigators only, and the names and confidential information of participants will not be provided to any third parties. All personal information will be considered confidential and used exclusively for study purposes. Document access will be restricted to those with specific permission, and the electronic dataset will only be accessed through unique usernames and passwords.

To further ensure confidentiality, study results will be published as aggregated data, which prevents the identification of individual participants. These data will be published for academic and scientific purposes only. All data will be managed according to the Brazilian General Personal Data Protection Law (Law No. 13709 of August 14, 2018) to safeguard the privacy of participants.

### Dissemination

To ensure transparency and dissemination of the study results, the investigators will present the complete results at relevant scientific meetings and conferences. Additionally, the study results will be published in peer-reviewed journals, following the guidelines and regulations set by the International Committee of Medical Journal Editors. The choice and selection of journals for publication will be made by the steering committee, based on the quality and relevance of the journal, as well as the potential impact of the study results on the scientific community. The study investigators will ensure that all publications clearly state the funding source and the role of the sponsors, if applicable. Finally, the study investigators will ensure that all publications accurately reflect the results obtained from the study and will take steps to address any potential conflicts of interest.

### Data sharing

Authors encourage data sharing and open access to promote transparency and reproducibility of research. Requests for data access should be submitted to the corresponding author and will be evaluated by the steering committee. Any data sharing will comply with the General Personal Data Protection Law (Law No. 13709 of August 14, 2018) and any applicable ethical and legal regulations. In case of data sharing, appropriate measures will be taken to ensure the confidentiality of the participants’ personal information.

## Discussion

The study aims to address a significant gap in the literature by investigating the impact of moderate-to-severe COVID-19 hospitalization on long-term quality of life, by conducting a rigorous observational study with a large sample size and long follow-up period. While there is scientific plausibility that COVID-19 severity may be associated with long-term quality of life, there is currently limited data available. Previous studies have been limited by small sample sizes and short follow-up periods, such as those with only three months of follow-up.^25^The recent multicenter observational Post-hospitalization COVID-19 (PHOSP-COVID) study evaluated hospitalized COVID-19 survivors five months after discharge and found that cardiovascular disease was the most common comorbidity (42%).^26^The authors of the study also found that only 28% of participants considered themselves fully recovered, and the predictors of lack of recovery or symptom persistence were female sex, white ethnicity, two or more comorbidities, and COVID-19 severity (based on the WHO scale, which includes mechanical ventilation or ECMO requirement during hospital stay).^24^The Coalition VII, a prospective multicenter cohort study that enrolled 1508 patients hospitalized with suspected or confirmed COVID-19, showed that patients who required mechanical ventilation during hospitalization had lower one-year quality of life than those who did not.^14^By conducting a rigorous observational study with a large sample size and a long follow-up period, covering the post-vaccination period and several predominant SARS-CoV-2 variants, the current study aims to further elucidate the long-term impact of moderate-to-severe COVID-19 hospitalization on quality of life.

Population-based data from Brazil indicate an increase in cardiovascular mortality during the first year of the COVID-19 pandemic.^27^This is likely due to the virus affecting the cardiovascular system through multiple mechanisms, such as microvascular dysfunction, oxygen supply-demand mismatch, direct myocardial injury, and cardiomyocyte toxicity.^28^These mechanisms in the acute phase of COVID-19 may contribute to long-term cardiovascular complications.^10 , 28^Therefore, it is crucial to evaluate and monitor patients for post-acute cardiovascular sequelae of SARS-CoV-2 infection to identify those at risk and provide appropriate treatment for secondary cardiac disease, including heart failure.^9 , 12 , 29^However, the actual burden of long COVID on cardiovascular health remains unknown, and further research is needed to determine its impact. Our study aims to highlight not only the prevalence of cardiovascular symptoms but also their outcomes.

The study has several strengths, including its prospective multicenter design with a large sample size of moderate-to-severe COVID-19 survivors followed for 12 months. However, several limitations should be acknowledged. For example, the association of long-term symptoms with prior COVID-19 may be subject to bias due to various factors, such as subjectivity in disease severity, personal health perceptions, and sociocultural factors related to the pandemic. Moreover, since no additional evaluations or complementary tests will be performed to exclude other causes of these symptoms, they may be erroneously attributed to previous SARS-CoV-2 infection.

It should be noted that the study planning and protocol were completed in November 2021, with recruitment scheduled to begin in December 2021. However, the steering committee reconsidered the study duration based on the number of cases in the population and the number of participants enrolled. The follow-up telephone calls commenced in March 2022, and the one-year follow-up is expected to be completed by April 2024.
